# Beyond Scientism and Skepticism: An Integrative Approach to Global Mental Health

**DOI:** 10.3389/fpsyt.2015.00166

**Published:** 2015-11-23

**Authors:** Dan J. Stein, Judy Illes

**Affiliations:** ^1^MRC Unit on Anxiety and Stress Disorders, Department of Psychiatry, Groote Schuur Hospital, University of Cape Town, Cape Town, South Africa; ^2^National Core for Neuroethics, Division of Neurology, Department of Medicine, University of British Columbia, Vancouver, BC, Canada

**Keywords:** neuroethics, global mental health, scientism, skepticism, causal mechanisms

## Abstract

The global burden of disorders has shifted from infectious disease to non-communicable diseases, including neuropsychiatric disorders. Whereas infectious disease can sometimes be combated by targeting single causal mechanisms, such as prevention of contact-spread illness by handwashing, in the case of mental disorders multiple causal mechanisms are typically relevant. The emergent field of global mental health has emphasized the magnitude of the treatment gap, particularly in the low- and middle-income world and has paid particular attention to upstream causal factors, for example, poverty, inequality, and gender discrimination in the pathogenesis of mental disorders. However, this field has also been criticized for relying erroneously on Western paradigms of mental illness, which may not be relevant or appropriate to the low- and middle-income context. Here, it is important to steer a path between scientism and skepticism. Scientism regards mental disorders as essential categories, and takes a covering law approach to causality; skepticism regards mental disorders as merely social constructions and emphasizes the role of political power in causal relations. We propose an integrative model that emphasizes the contribution of a broad range of causal mechanisms operating at biological and societal levels to mental disorders and the consequent importance of broad spectrum and multipronged approaches to intervention.

## Introduction

In recent decades, there has been a shift from infectious disease to non-communicable diseases throughout the world. Mental, neurological, and substance use disorders are already the largest contributor to the burden of disease; these prevalent, chronic, and costly disorders now account for 22% of disablilty adjusted life years (DALYs) from all medical causes in those aged 15–49 (1). Furthermore, forecasts indicate that in the foreseeable future they will become even more central to global public health, with the World Economic Forum predicting that neuropsychiatric disorders will comprise the largest costs of chronic, non-communicable diseases globally in the next two decades (2).

Increased recognition of the burden of neuropsychiatric disorders has given impetus to the emergence of the discipline of global mental health (3). Additional key considerations are that interventions for mental disorders impact positively on individual well-being and country development and are highly cost-efficient, but that neuropsychiatric disorders are often underdiagnosed and undertreated, with the treatment gap particularly large in low- and middle-income countries. Furthermore, this treatment gap is a human rights issue; levels of stigmatization of people living with mental illness are too high, and levels of mental health literacy are too low in communities, clinicians, and policy makers (4, 5).

Clinical and research work on neuropsychiatric disorders raises a number of conceptual and ethical questions, many of which are relevant to the field of global mental health (6). In considering some of these conceptual and ethical questions, we have argued that clinicians and researchers should steer a course between a scientism that regards mental disorders as natural kinds, and a skepticism that views all mental disorders as mere sociocultural constructions (7–9). Integrative approaches are needed to address fully the complex reality of mental disorders. In this commentary, we discuss this view in relation to global mental health, considering in turn issues of diagnosis, pathogenesis, and intervention (Table 1).

**Table 1:** **Moving beyond scientism and skepticism in global mental health: integrative approaches to diagnosis, pathogenesis, and intervention**.

	Scientism	Skepticism	Integrative
Diagnosis	Diagnostic systems rely on essentialist categories or natural kinds. Assessment systems will be ultimately be supported by data on endophenotypes	Mental illness is expressed and experienced differently in different sociocultural contexts. Symptoms vary from time to time and place to place	Mental illness is a complex reality. Nosologies are theory bound and value laden, but may improve as the relevant science and debate advance
Pathogenesis	May approach causality in terms of covering laws. May focus on a single set of associations, such as those which characterize the health care system	May emphasize the role of sociocultural values and powers in explanations. May focus on differences in conceptualization of disorders across history and geography	Emphasizes that a broad range of factors are involved in the pathogenesis of mental disorders, with causal mechanisms operating at multiple interacting levels
Intervention	May take a single-bullet approach, looking for focused interventions, whether biological or community focused that will target the essence of the disorder	May emphasize that interventions reflect local values and powers. Both biological and community-focused interventions reinforce existing societal structures	Incorporates a range of insights about the nature of mental disorders, and targets a broad range of factors involved in their pathogenesis, including biological and social ones

## Global Mental Health and Diagnosis

Global mental health has emphasized that mental disorders are prevalent and associated with significant suffering, impairment, and socioeconomic costs. Thus, for example, data from the World Mental Health Surveys have emphasized that mental disorders are more impairing than physical disorders, but are less likely to be diagnosed and treated (10). While such conclusions are pertinent around the globe, in low- and middle-income countries, a lack of resources is particularly likely to exacerbate the treatment gap. These sorts of data provide an important foundation for the rallying cry of global mental health that there is no health without mental health (11).

Nevertheless, global mental health has also come under fire for its emphasis on these sorts of data. In particular, critics have argued that the field relies erroneously on Western paradigms of mental illness, which may not be relevant or appropriate to the low- and middle-income context (12). Such constructs run the risk of ignoring how symptoms vary from time to time and from place to place, and of downplaying the complex ways in which illnesses are expressed and experienced differently in different sociocultural contexts (13). Indeed, Jacob and Patel have emphasized that global mental health needs new diagnostic approaches, a view that is perhaps partially consistent with attempts in clinical neuroscience to reformulate approaches to evaluation of neuropsychiatric disorders (14, 15).

At the same time, international classification systems have significant clinical advantages, and there are currently no viable alternatives in practice. We would, therefore, argue that although it is clearly important to recognize the limitations of current psychiatry nosology and biopsychosocial models (16, 17), we ought to be wary of unrealistic expectations of such approaches (18). For example, medicine does not require that its diagnostic systems are essentialist in nature; rather medical syndromes provide clinicians with a practical set of tools for assessing patients. Rather than insisting that assessment systems will ultimately be supported solely by data on endophenotypes – intermediate phenotypes with high heritability – we can also ask that more work is also needed on exophenotypes, such as societal, structural, and other upstream contributors to disease and illness (19), and their intersections.

## Global Mental Health and Pathogenesis

Although infectious diseases may involve a range of biological and psychosocial factors, it is sometimes possible to combat these conditions by targeting single causal mechanisms. Locating the geographic source of a cholera epidemic, employing handwashing to decrease bacterial transmission, developing vaccines to prevent polio and smallpox, and using mosquito nets to prevent malaria have been seminal exemplars of success for public health. In contrast, global mental health has had to contend with multiple upstream factors that impact mental disorders: poverty, inequality, gender discrimination, and more.

At the same time, any emphasis of global mental health on only one set of causal factors can potentially be problematic. Some research priority setting exercises have indicated that global mental health should focus primarily on health systems research, for example, and should pay less attention to the biological causes of mental disorders (20). This is consistent with a criticism of global mental health which emphasizes that it is ironic that a field that purports to be concerned with a broad range of socioeconomic factors relies on neuroessentialist DSM-5 categories. After all, key considerations for Western-based typologies of illness are that they have diagnostic validity, that disorders demonstrate high heritability, or that they predict response to interventions such as pharmacotherapy.

Our own view is that there are important opportunities at the intersection of global mental health and clinical neuroscience in addressing the pathogenesis of mental disorders (21). There has been significant progress on understanding how nature and nurture intersect to create vulnerabilities for mental disorder, and indeed in recognizing how multiple levels of causal factors contribute to these conditions (7, 22). We have previously noted, for example, that while basic neuroscience has shed a great deal of insight into addiction, a full understanding of substance use disorders requires the psychological and social levels to be included (8, 9). Only a comprehensive and integrative perspective will allow an understanding of complex phenomena, such as decreased voluntary control in addictive disorders (23).

## Global Mental Health and Intervention

Global mental health has focused on task shifting and implementation science. This is certainly important in the context of resource-limited settings, where there are simply not enough trained professionals to deliver interventions, where health systems have systemic problems, and where there is growing evidence that non-specialized community workers can make a real impact (3). Indeed, some of the concerns of global mental health mirror those of the solution-oriented bent of neuroethics; there is a focus on efforts to improve wellness, on the importance of human rights, and on an empirical approach to optimizing interventions (6).

At the same time, there are potential criticisms of the focus of global mental health on communities, task shifting, and implementation science. Sartorius and colleagues, for example, have noted that in many parts of the globe, communities have changed in significant ways and are no longer able to provide the support that those with serious mental illness need and deserve (24). Furthermore, some tasks simply cannot be shifted, and we need to focus at times rather on novel biological treatments (25) or on increasing resources; it is crucial that in attempting to strengthen resource-limited systems, we do not simply institutionalize mechanisms that can only work in impoverished systems.

Again, we would argue for an integrative approach. It is important to avoid a scientism which states that given that mental disorders are natural kinds, they will ultimately succumb to single-bullet biological interventions (26). At the same time, we do not want to fall to prey to a skepticism that indicates that interventions should be entirely focused on changing the way in which disorders are conceptualized and labeled by society, or that they should be limited to community practices. We need an integrative approach to intervention that incorporates a range of insights about the nature of mental disorders and that targets a broad range of factors involved in their pathogenesis, including psychobiological factors and community processes.

## Conclusion

We have argued elsewhere that it is important to avoid neuroreductionism and to emphasize instead that mental and substance use disorders require an understanding of psychosocial factors. Put differently, it is important to steer a path between scientism, which regards mental disorders as essential categories and takes a covering law approach to causality, and skepticism, which regards mental disorders merely as social constructions and reduces causality to considerations of political power. Here, we have applied these arguments to the newly emergent field of global mental health, considering issues relevant to diagnosis, pathogenesis, and treatment (Table 1), and emphasizing that a broad range of causal mechanisms operating at biological, psychological, and societal levels, and at the interactions between these levels, contribute to mental disorders, and that clinical interventions and research practices must match this complexity.

## Conflict of Interest Statement

In the past 3 years, DS has received research grants and/or consultancy honoraria from AMBRF, Biocodex, Cipla, Lundbeck, National Responsible Gambling Foundation, Novartis, Servier, and Sun. JI has no conflicts to declare.
